# *TWIST1* Gene: First Insights in *Felis catus*

**DOI:** 10.2174/138920210791110933

**Published:** 2010-05

**Authors:** Cláudia S. Baptista, Estela Bastos, Sara Santos, Ivo G. Gut, Henrique Guedes-Pinto, Fátima Gärtner, Raquel Chaves

**Affiliations:** 1 Department of Veterinary Clinics, Institute of Biomedical Sciences Abel Salazar (ICBAS), University of Porto, Largo Prof. Abel Salazar, 2, 4099-003 Porto, Portugal; 2 Institute for Biotechnology and Bioengineering, Centre of Genetics and Biotechnology, (IBB, CGB-UTAD), University of Trás-os-Montes and Alto Douro, Quinta de Prados, 5001-801 Vila Real, Portugal; 3 CEA/DSV/IG-Centre National de Génotypage, Bâtiment G2, 2 rue Gaston Crémieux, CP 5721, 91057 Evry Cedex, France; 4 Department of Pathology and Molecular Immunology, Institute of Biomedical Sciences Abel Salazar (ICBAS), University of Porto, Largo Prof. Abel Salazar, 2, 4099-003 Porto, Portugal; 5 Institute of Pathology and Immunology (IPATIMUP), University of Porto, Rua Dr. Roberto Frias, s/n, 4200-465 Porto, Portugal

**Keywords:** *TWIST1* gene, *Felis catus*, comparative analysis, oncology.

## Abstract

*TWIST1* is thought to be a novel oncogene. Understanding the molecular mechanisms regulating the *TWIST1 *gene expression profiles in tumor cells may give new insights regarding prognostic factors and novel therapeutic targets in veterinary oncology. In the present study we partially isolated the *TWIST1* gene in *Felis catus* and performed comparative studies. Several primer combinations were used based on the alignments of homologous DNA sequences. After PCR amplification, three bands were obtained, purified and sequenced. Several bioinformatic tools were utilized to carry out the comparative studies. Higher similarity was found between the isolated *TWIST1* gene in *Felis catus* and *Homo sapiens *(86%) than between *Homo sapiens* and *Rattus norvegicus* or *Mus musculus* (75%). Partial amino acid sequence showed no change in the four species analyzed. This confirmed that coding sequences presented high similarity (~96%) between man and cat. These results give the first insights regarding the *TWIST1* gene in cat but further studies are required in order to establish, or not, its role in tumor formation and progression in veterinary oncology.

## INTRODUCTION

The *TWIST1* gene was originally identified in *Drosophila melanogaster* where its activity was found to be crucial for the establishment of dorsoventral pattern and mesoderm formation of the embryo. This gene encodes a protein called Twist-1, a highly conserved transcription factor that belongs to the family of basic helix–loop–helix (bHLH) proteins [1-3].

In human, this gene (GenBank: NC_000007.13 and Ensembl: ENSG00000122691) has a length of 2188 bp and is organized in two exons (1002 bp and 647 bp, respectively, exon 1 and exon 2) and one intron (539 bp). The first exon contains an open reading frame encoding a 202 amino acid protein (GenPept: AAC50930.1) being, currently, only one transcript characterized (GenBank: NM_000474 and Ensembl: ENST00000242261). In human, the *TWIST1 *gene maps to 7p21.1 and is expressed in the head and limb buds in developing embryos [4]. In adult human, its expression is predominantly detected in mesodermally derived tissues [5].

Several studies suggest that the *TWIST1* gene is a novel oncogene that is associated with tumor formation and/or metastatic spread. In fact, besides the regulation of the embryonic morphogenesis, the Twist-1 protein has multiple functions during tumorigenesis, including inhibition of cell safeguard programs, altered cell–cell adhesion, and deregulation of differentiation, contributing to increased cell survival and invasion [5-9].

Mutations on the coding region of the human *TWIST1* gene, leading to haploinsufficiency, have been identified in Saethre–Chotzen syndrome (SCS) [10, 11], in some cases of Baller-Gerold syndrome [12] and in human pediatric osteosarcomas [13]. Recently, Sahrin and colleagues [14] demonstrated an increased risk of breast cancer in women with Saethre-Chotzen syndrome, suggesting that germline mutations in *TWIST1* may also predispose to breast cancer.

*TWIST1* overexpression was reported in a variety of solid cancers including breast, prostate and gastric carcinomas, melanomas, osteosarcomas, rhabdomyosarcomas, as well as in Sezary syndrome [6, 8, 13, 15-20].

Besides *Drosophila* and human, members of the *TWIST* family have also been identified in different species such as frog [21], mouse [22], leech [23], zebrafish [24], lancelet [25], nematode [26], jellyfish [27] and chicken [28]. To the best of our knowledge, this gene has never been described in *Felis catus*.

In the present work we achieved the partial isolation of *TWIST1* gene in cat. As far as we know, *TWIST1* has never been previously isolated in cat. We accomplish this purpose in order to carry out comparative studies between *Felis catus* and other species, namely *Homo sapiens*, and to perform *in silico* mapping of this gene in cat. We present a new approach than can be used to *in silico* gene map, and that use several tools that result from the cat and canine genomes sequencing efforts, that is, sequence data and radiation hybrid maps. We believe that this knowledge is a starting point that may permit the research of the molecular mechanisms regulating *TWIST1* gene expression in veterinary oncology.

## MATERIAL AND METHODS

### Genomic DNA Extraction and PCR Amplification

Blood samples from six different cats were used as the biological material for DNA extraction. After owners consent, blood was collected by venipuncture and stored in tubes with heparin as anticoagulant. DNA extraction was performed from 200 μL of total blood using automatic equipment (Quickgene-810, Fujifilm), and the Quickgene Whole Blood Kit S (Fujifilm) according to instructions of the manufacturer. In order to analyze the integrity of the genomic DNA extracted all samples were subjected to a 1.5% agarose gel electrophoresis. The quantity and quality of the DNA extracted was also determined in a NanoDrop ND-1000 spectrophotometer (NanoDrop Technologies).

In order to isolate the *TWIST1* gene from *Felis catus* genomic DNA, primers were designed based on the alignments of homologous DNA sequences, from species where this gene was already identified, *Homo sapiens* (GenBank NC 000007.12) and *Canis familiaris *(GenBank NC 006596.2).

The different primer combinations and corresponding sequences are shown in Table **1**. For combination **1**, DNA was amplified after 95°C denaturation for 5 min with 35 cycles of 95ºC for 30 s, 65ºC for 30 s, 72°C for 30 s and a 72°C final extension step for 10 min. The 25 μL PCR reaction mix contained: 1.5 μL each primer (100 ng/μL), 1.5 μL MgCl2 at 25 mM, 2 μL dNTP mix (2.5 mM), 2 μL DNA (78 ng/μL), 0.5 μL (5 U/μL) Taq DNA polymerase (Fermentas), 2.5 μL buffer and 13.5 μL water. For primers combination **2** and **3**, the PCR temperature cycle conditions and concentrations were identical to **1** except for the annealing temperature which was 60ºC instead of 65ºC.

All the amplifications were performed on a T-Personal Thermal Cycler (Biometra) and the PCR products were analyzed by electrophoresis in 1.5% agarose gel, stained with ethidium bromide, visualized under UV light and digitally recorded (UviDoc).

The PCR fragments were excised from the agarose gel and purified, according to the procedure described by Geneclean II Brief Protocol: Purifying DNA (QBiogene).

### RNA Extraction and RT-PCR Amplification

Necropsy of a cat with several traumatic lesions due to a car accident was immediately performed after euthanasia for fresh tissue sampling. Other than the skull and cervical vertebral column, no significant internal lesions were observed. Macroscopically normal tissue samples such as skin, liver, spleen, lung, kidney, testicles and bone marrow were stored (-80ºC) in a RNA stabilization solution (RNA Later Tissue Collection, Ambion). Total RNA was extracted with the standard Trizol method (Invitrogen) and quantified by spectrophotometry using the NanoDrop ND-1000 (NanoDrop Technologies). The total reaction volume for cDNA synthesis and amplification (one step reaction) was 20 μL including: 1 µL of RNA extracted from testicles (1 µg/µL), 8 µL of the One-step RT-PCR pre-mix (Intron), 1 μL of each primer (5’ GAGCCCGCAGTCGTACGAG 3’ and 5’ CTCTGGA GGACCTGGTAGAGGA 3’; 100 ng/μL) and 9 μL of water. cDNA was amplified after reverse transcription at 45ºC for 30 min, 94°C denaturation for 5 min with 29 cycles of 94ºC for 30 s, 57ºC for 30 s, 72°C for 30 s, with a 72°C final extension step for 5 min. This amplification was performed on a T-Personal Thermal Cycler (Biometra) and the RT-PCR product was analyzed in a 1.5% agarose gels stained with ethidium bromide and visualized with an ultraviolet transilluminator UviDoc.

The cDNA was excised from the agarose gel and purified with the Geneclean II Brief Protocol: Purifying DNA kit (QBiogene).

### Sequencing of the Amplified Products

PCR samples were sequenced in both directions and sequence analysis was performed using the bioinformatic resource at the NCBI: Basic Local Alignment Search Tool (BLAST) and the Vector NTI software (Invitrogen Life Technologies). The feline *TWIST1* DNA sequence and the *TWIST1* cDNA sequence have been submitted to GenBank with the respectively accession number GQ167299 and GQ167300.

### Phylogenetic Analysis

For the phylogenetic and evolutionary analysis of the molecular sequence data we used Phylemon [29], an online platform that integrates a suite of more than 20 different tools.

The largest sequenced fragment (960 bp) (fragment 3 from Table **1**) was aligned with *TWIST1* gene from *Homo sapiens *(GenBank NC_000007.12), *Macaca mulatta *(GenBank NC_007860.1),* Pan troglodytes *(Ensemble release 47: ENSPTRG00000018960, Contig 5.948 and Contig 5.949), *Mus musculus* (GenBank NC_000078.5),* Rattus* *norvegicus* (GenBank NC_005105.2),* Bos Taurus* (GenBank NC_007302.2) and *Gallus gallus* as the outgroup species (Ensemble release 47: ENSGALT00000010219, Contig 16422).

We utilized the ClustalW v1.83 program [30] for the alignment of the previously mentioned multiple sequences. Basic maximum likelihood (ML) analyses of the DNA sequence data were provided with the DnaML algorithm of the PHYLIP package (version 3.65). Details of the algorithm are published in the paper by Felsenstein and Churchill [31].

In order to confirm the phylogram we used the MEGA4 program [32] that utilizes Maximum Composite Likelihood (MCL) method for estimating evolutionary distances between DNA sequences. The phylogeny reconstruction analysis was performed with the Neighbor-Joining method and the Nucleotide: Maximum Composite Likelihood substitution model.

### Protein Prediction

For protein prediction of the 960 bp fragment of *TWIST1* gene, we used the tool TranSeq from EMBOSS (European Molecular Biology Open Software Suite) [33]. This tool translates nucleic acid sequences to the corresponding peptide sequence. We used frame 2 after analysing the six possible reading frames. Simultaneously, in order to confirm the result, we used multiple alignments of the eight sequences performed by the ClustalW program, described on the previous section. We converted the multiple alignment into the multi sequence format (msf) used by GenDoc program, a multiple sequence alignment editor developed by Nicholas *et al*. [34]. The protein prediction was confirmed on the Genedoc program allowing to simultaneously edit the aligned sequences and execute the translation of the coding region into the amino acid sequence.

### Gene Localization

The *TWIST1* gene was *in silico* physically mapped in *Felis catus*. In order to perform a comparative analysis we also mapped this gene in the following species: *Homo sapiens*, *Pan troglodytes*, *Macaca mulatta*, *Bos taurus*, *Gallus gallus*, *Mus musculus*, *Rattus norvegicus* and *Canis familiaris*.

The *in silico* analysis of the largest sequenced fragment (960 bp), partial sequence of the *TWIST1* gene, was primarily performed using the “MultiBlast Analysis” (ENSEMBL Genome Browser). All the results obtained were confirmed using the “SyntenyView” (ENSEMBL Genome Browser). Since the cat karyotype is not available in the ENSEMBL Genome Browser, the physical map of this gene was established with the Genome Browser for *Felis catus*, the Genome Annotation Resource Fields – GARFIELD [35], the initial sequence and comparative analysis of the cat genome published by Pontius *et al*. [36], and the radiation hybrid maps from Murphy *et al*. [37] and Davis *et al*. [38]. We also used the dog map information in order to improve the localization of the *TWIST1* gene in the cat chromosome. We used the 4249 marker FISH/RH map of the canine genome for chromosome 14 from Breen *et al*. [39] and the FISH/RH maps available from the same author at http://cvm.ncsu.edu/mbs/ breen/dog_map.htm.

The genome versions used in ENSEMBL Genome Browser were: *Homo sapiens *(NCBI36), *Pan troglodytes *(CHIMP2.1), *Macaca mulatta *(MMUL1.0), *Bos taurus *(BTAU_4.0), *Gallus gallus *(WASHUC2), *Mus musculus *(NCBI m37), *Rattus norvegicus *(RGSC 3.4), *Canis familiaris *(CanFam2.0) and *Felis catus* (CAT).

## RESULTS

### Genomic DNA Extraction and PCR Amplification

The DNA samples obtained from 200 μl of blood using Quickgene Whole Blood Kit S (Fujifilm) showed a satisfactory concentration (between 67.66 ng/μL and 88.57 ng/μL, with a total yield of 200 μL). The DNA integrity evaluated by agarose gel electrophoresis and by spectrophotometry was satisfactory (OD_260_/OD_280 _=1.91).

In order to amplify the *TWIST1* gene from cat genomic DNA extracted from 6 blood samples, we synthesized multiple primers based on the homologous sequences from *Homo sapiens* (GenBank NC_000007.12) and *Canis familiaris* (GenBank NC_006596.2). To successfully achieve this goal several primers combinations were used (Table **1**) and, for each sample, the obtained fragments have, approximately, the following lengths: **1**=300 bp; **2**=800 bp and **3**=1000 bp (**1a, 1c**).

### RNA Extraction and RT-PCR Amplification

**Fig. (1):**
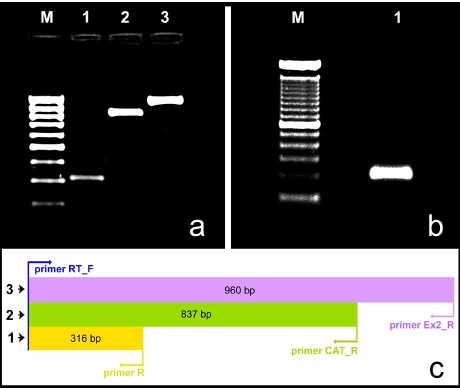
(**a**) Electrophoresis of the PCR products obtained from cat gDNA extracted from 200 μl of blood in a 1.5% agarose gel. Three bands of different sizes were obtained. Lane M: Molecular marker (Gene Ruler 100 bp DNA Ladder, Fermentas). Lanes 1-3: Amplified products with approximately 300, 800 and 1000 bp; (**b**) Electrophoresis of the RT-PCR product in a 1.5% agarose gel. Lane M: Molecular marker (Mass Ruler DNA Ladder Mix, Fermentas). Lane 1: RT-PCR fragment with approximately 200 bp. (**c**) Scheme for the sequencing of *Felis catus TWIST1* gene with the identification and localization of primers and the respective product length.

To verify if the *TWIST1* gene in *Felis catus* was a coding gene and, if so, in order to localize the coding region, we performed reverse-transcriptase PCR amplification. Total RNA extracted from testicles, liver and bone marrow with the standard Trizol method (Invitrogen) presented a good quantity (281.5 ng/μL, 189 ng/μL and 210.5 ng/μL respectively) and quality evaluated by spectrophotometry (OD_260_/OD_280 _= 1.86, 1.87 and 1.83, respectively). Using primers 5’-GAGCCCGCAGTCGTACGAG-3’ and 5’-CTCTGGAGGACCTGGTAGAGGA-3’, designed based in homologous sequences from *Homo sapiens* (GenBank NC_000007.12) and *Canis familiaris* (GenBank NC_006596.2), in each sample, we amplified a fragment with around 200 bp (Fig. **1b**).

### Sequencing of the Amplified Products

After PCR amplification using the multiple primers combinations evidenced in Table **1** and Figs. (**1a** and **1c**), the obtained fragments were excised from the agarose gel, purified and directly sequenced in both directions. After thorough analysis of the 36 sequencing reactions, we isolated 960 bp of the feline *TWIST1* gene DNA. This sequence is shown in Supplementary Fig. (**1**), where it is aligned with the human sequence. Strong similarity (86%) was found between the human sequence and the amplified feline DNA. We can observe major differences in the intron, whereas exon 1 and exon 2 are highly conserved.

To analyze mRNA encoded by the *TWIST1* gene, primers were designed on the feline DNA putative coding region, identified on the basis of similarity between the feline (Supplementary Fig. **1**) and the human DNA sequence. cDNA was obtained from retro-transcription of total RNA isolated from cat testicle. This amplification, allowed us to isolate a fragment with around 200 bp which was also excised from an agarose gel, purified and sequenced in both directions. After sequence evaluation, the resulting 201 bp of feline cDNA was subject to BLAST analysis. The information confirmed that we isolated the cat *TWIST1* gene cDNA. Using the ClustalW program, we aligned this sequence (Supplementary Fig. **1**, underline 201 bp sequence) with the partially isolated predicted coding region (Supplementary Fig. **1**, blue 316 bp sequence) and we concluded that the isolated cDNA is 100% included in the predicted coding region of the *Felis catus TWIST1* gene.

### Phylogenetic Analysis

The 960 bp sequence from cat *TWIST1* gene was aligned with homologous sequences from *Homo sapiens*, *Macaca mulatta*, *Pan troglodytes*, *Mus musculus*, *Rattus norvegicus*, *Bos taurus* and *Gallus gallus* in order to obtain the phylogram presented in Fig. (**2**). The results displayed by the phylogenetic analysis allowed us to conclude that there is a higher similarity between the homologous sequences from *Homo sapiens* and *Felis catus* (86%) than similarity between *Homo sapiens* and *Rattus norvegicus* or *Mus musculus* (75%). We also found a high similarity (96%) between *Homo sapiens* and *Felis catus* regarding the 316 bp (partial exon 1) fragment. In fact, we detected a higher similarity between cat and man for the sequence analyzed when comparing with other animal models, as the classical rat and mouse.

### Protein Prediction

The deduced partial amino acid sequence of the Twist-1 protein in all species analyzed was generated by the Genedoc program, starting from the Phylemon platform. The maximal length of the partial coding region isolated from cat genomic DNA has 316 bp (Fig. **1c** and Table **1**, primer combination 1) and 104 deduced amino acids from a predicted total of 202 amino acids. The results displayed were confirmed comparing our amino acid sequence with known amino acid sequences from GenBank database, from *Homo sapiens *(GenBank NC_000007.12), *Rattus* *norvegicus* (GenBank NC_005105.2),* Mus musculus* (GenBank NC_000078.5),* Macaca mulatta *(GenBank NC_007860.1),* Pan troglodytes *(Ensemble release 47: ENSPTRG00000018960, Contig 5.948 and Contig 5.949),* Bos Taurus* (GenBank NC_007302.2) and *Gallus gallus* (Ensemble release 47: ENSGALT00000010219, Contig 16422). The multiple alignments were performed by the ClustalW program (Fig. **3**) and they show no change in all the species analyzed, except for *Gallus gallus* (the outgroup species) where 13 amino acids were modified. Between *Homo sapiens* and *Felis catus* we detected 8 transitions and 2 transversions meaning that the conservation at the protein level is different from the scenario observed at the genomic level.

### Gene Localization

The results regarding the *in silico* physical mapping of the *TWIST1* gene in *Felis catus, Canis familiaris, Homo sapiens, Pan troglodytes, Macaca mulatta, Mus musculus, Rattus norvegicus, Bos taurus* and *Gallus gallus* species are presented in Fig. (**4**).

The *in silico* analysis of the largest sequenced DNA fragment from the cat *TWIST1* gene (i.e. 960 bp DNA fragment) was blasted on the genomes from the species in analysis, using the “MultiBlast Analysis” tool from the ENSEMBL Genome Browser. This analysis allowed the physical mapping of the cat *TWIST1* partial sequence on the following chromosome (chr) species (Fig. **4c**): *Canis familiaris* chr 14, *Homo sapiens* chr 7p21.1, *Pan troglodytes* chr 7, *Macaca mulatta* chr 3, *Mus musculus* chr 12B2, *Rattus norvegicus* chr 6q16 and *Bos taurus* chr 4. Moreover, in all analysis, and for each chromosome, it was possible to determine the localization of the predicted gene in terms of Mb with a higher precision (Fig. **4c**). All the results obtained were confirmed using the “SyntenyView” tool from the ENSEMBL Genome Browser what allows the search of the syntenic segments between these genomes (Fig. **4c**). The *TWIST1* gene was mapped to chr 2 in *Gallus gallus*; however, this analysis was only possible through the physical mapping of the cat *TWIST1* gene made in the human genome using the “view synteny regions” tool in the “contigview” menu. Therefore, the localization of the predicted gene in terms of Mb in this chromosome was not as accurate (see Fig. **4c**) as for the other genomes.

Finally, the cat *TWIST1* gene was physically mapped to chr A2 (q21.3) in the cat karyotype (Fig. **4b**). In this case we used the Genome Browser for *Felis catus*, the Genome Annotation Resource Fields – GARFIELD [35], the initial sequence and comparative analysis of the cat genome published by Pontius *et al*. [36], and the radiation hybrid maps from Murphy *et al*. [37] and Davis *et al*. [38]. The simple search of the *TWIST1* gene in the cat genome showed the chromosome A2 involved (see Fig. **4b**), with the fine localization in terms of Mb on this chromosome. However, this analysis does not permit to physically map the gene in the A2 ideogram (physical mapping at band level). In order to accomplish this task, we used the cat radiation hybrid maps [37-38] and the 4249 marker FISH/RH map of the canine genome for the 14 chromosome [39] and the FISH/RH maps available at http://cvm.ncsu.edu/mbs/breen/dog_map.htm. With this comparative analysis it was possible to *in silico* map the *TWIST1* gene to the q21.3 band from cat chromosome A2 (Fig. **4c**).

## DISCUSSION

It is commonly accepted that naturally occurring cancers in dogs and cats offer a unique opportunity as models for human cancer biology and translational cancer therapeutics. Several aspects contribute to the advantages of the companion animal model such as the relatively high incidence of some cancers when compared with humans, similar biological behavior with faster rate of progression, large body size that allow imaging studies as well as surgical interventions, comparable responses to cytotoxic agents and radiation therapy, shorter overall lifespan and similar environmental risk factors. It is also important to consider that animal trials are generally much more economical to run than human trials [40-42]. In this work we present evidence that *TWIST1* may be targeted in new studies to understand its role in feline oncology and that, potentially, *Felis catus* could be a very interesting animal model to study human disease, at least regarding this gene.

To the best of our knowledge, *TWIST1* has never been previously isolated in cat. Therefore, in order to accomplish this purpose and perform comparative studies, several primer combinations were designed to successfully obtain the partial amplification of the *TWIST1 *gene in this species. The segments of interest had a high GC content making this task somewhat challenging, but we were able to successfully isolate 960 bp of the cat *TWIST1* gene, namely 358 bp of the exon 1 (including 316 bp of the coding region), 516 bp of the intron and 86 bp of the exon 2 (Fig. **5**). When compared to the homologous sequence in *Homo sapiens*, this isolated sequence presented major genomic differences in the non-conserved intronic region, as expected (80% similarity). Evolutionarily conserved regions, meaning exon 1 and exon 2, evidenced 96% similarity between both these species.

According to the ENSEMBL Genome Browser latest release 57 (March 2010), in cat, the homologous region with respect to the human *TWIST1* gene predicts a novel pseudogene (ENSFCAG00000006288). These genes are defined as genomic DNA sequences that bear significant homology to functional genes but have lost their potential as DNA templates for functional products [43, 44]. However, in this research work, the RT-PCR results demonstrated that the feline *TWIST1* gene is a transcribed gene, evidencing, at least, one transcript. However, additional studies are required in order to study its expression profile in normal and tumor cells, in this species.

**Fig. (2):**
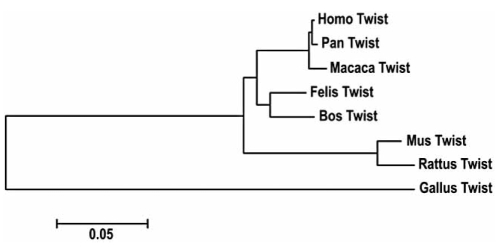
Phylogram constructed using the alignment between the isolated 960 bp sequence from cat *TWIST1* gene and homologous sequences from *Homo sapiens, Macaca mulatta, Pan troglodytes, Mus musculus, Rattus norvegicus, Bos taurus and Gallus gallus.*

**Fig. (3):**
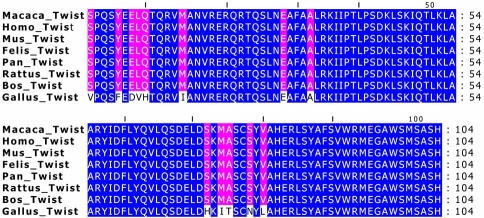
For all species analyzed (*Homo sapiens, Rattus norvegicus, Mus musculus, Macaca mulatta, Pan troglodytes, Bos Taurus and Gallus gallus*) the partial amino acid sequence of the Twist-1 protein was deduced using the Genedoc program, starting from the Phylemon platform, and the multiple alignments were performed using the ClustalW program. We can observe 104 amino acids (316 bp of the coding region isolated in cat) from a predicted total of 202 amino acids (coding region with 609 bp). Only the outgroup species (*Gallus gallus*) evidences 13 different amino acids.

**Fig. (4):**
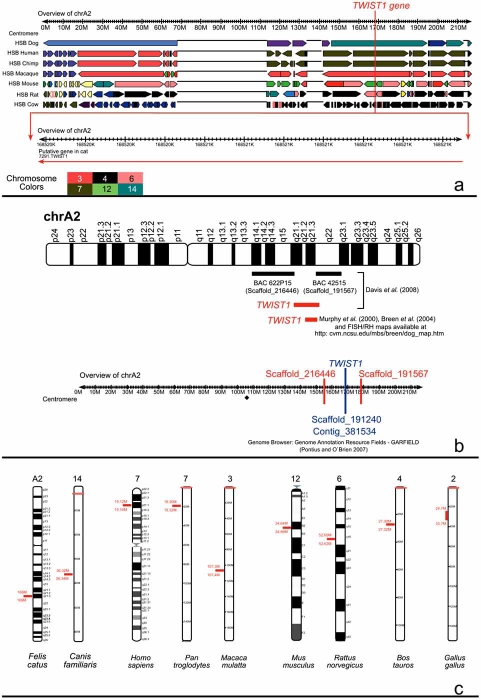
Physical mapping of the *TWIST1* gene in *Felis catus, Canis familiaris Homo sapiens, Pan troglodytes, Macaca mulatta, Mus musculus, Rattus norvegicus, Bos taurus and Gallus gallus species.* **(a)** *In silico* comparative analysis of the *TWIST1* gene with the Genome Annotation Resource Fields – GARFIELD [35]. **(b)** The *in silico* physical map of this gene was established with the Genome Browser for *Felis catus*, the Genome Annotation Resource Fields – GARFIELD [35], the initial sequence and comparative analysis of the cat genome published by Pontius *et al*. [36], and the radiation hybrid maps from Murphy *et al.* [37] and Davis *et al.* [38]. We also used the dog map information in order to improve the localization of the *TWIST1* gene in the cat chromosome. We used the 4249 marker FISH/RH map of the canine genome for chromosome 14 from Breen *et al.* [39] and the FISH/RH maps available from the same author at http://cvm.ncsu.edu/mbs/breen/dog_map.htm. **(c)** Chromosome ideograms showing the *TWIST1* gene localization in several species analysed in the present work.

**Fig. (5):**
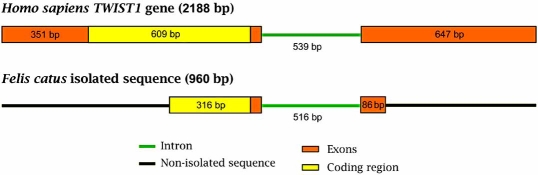
Schematic representation of the *Homo sapiens TWIST1* gene and the orthologous gene sequence isolated in the present work from *Felis catus* (960 bp in total). We can observe that we have isolated 358 bp of exon 1 (including 316 bp of the coding region), 516 bp of the intron and 86 bp of the exon 2.

The phylogenetic analysis performed with the alignment of largest cat DNA fragment from *TWIST1* gene and homologous sequences from several species (Fig. **2**), detected a higher similarity between the sequences of cat and man. This information is extremely important, suggesting the cat as an attractive model, at least for *TWIST1* gene studies, and that should be used instead of the classical animal models (e.g. the rat and mice). This data is supporting a growing body of evidence that propose alternative animal models, as the cat, relative to the classical ones. In fact, the evolutionary history of the genomes indicates great conservation between cat and human, with around 30 homologous segments separating both genomes. In contrast, genomes as the ones of rat, mouse and dog are four times more reshuffled than those of species retaining the more slowly evolving genomes (e.g. cat and human) [45, 46].

The partial amino acid sequence (104 aa from 202 aa, in total) inferred from the coding region within fragment 3 (Fig. **1c**), showed no change in all the species analyzed, except for *Gallus gallus* (the outgroup species) where 13 aminoacids were modified. This conservation at the protein level is different from the scenario observed at the genomic level. We detected 8 transitions and 2 transversions when comparing the coding fragment between *Homo sapiens* and *Felis catus*. All these sequence variations correspond to synonymous or silent mutations. Considering these evidences, it is encouraging to think that the ongoing studies regarding the *TWIST1 *gene in *Homo sapiens *can be extrapolated to *Felis catus*.

The *in silico* analysis presented allowed the physical map of TWIST1 gene to several species: Felis catus chr A2q21.3, *Canis familiaris* chr 14, *Homo sapiens* chr 7p21.1, *Pan troglodytes* chr 7, *Macaca mulatta* chr 3, *Mus musculus* chr 12B2, *Rattus norvegicus* chr 6q16 and *Bos taurus* chr 4. The importance of gene mapping in genetic clinical studies is well known for diagnosis purposes. In this paper we present a new approach that can be used to *in silico* gene map, and can use several tools that result from the cat and canine genomes sequencing efforts, that is, sequence data and radiation hybrid maps.

## CONCLUDING REMARKS

We believe that this investigation is the first to isolate the partial sequence of the *TWIST1* gene in the *Felis catus* and to perform comparative analysis between this oncogene in cat with other species, namely *Homo sapiens*. We observed that, regarding this gene, there is a higher similarity between cat and man than between man and other widely used animal models such as rat or mouse, and that *TWIST1* maps to the q21.3 band from cat chromosome A2. This work gives the first insights regarding the *TWIST1* gene in *Felis catus,* so in the future, we may contribute to the study of the molecular mechanisms affecting its expression profile in cat tumor cells.

## SUPPLEMENTARY MATERIAL

Supplementary material is available on the publishers Web site along with the published article.

## Figures and Tables

**Table 1:** Primer Combinations Used for the DNA Isolation of *Felis catus TWIST1* Gene

Forward sense strand	Reverse sense strand	Combination
RT-F: 5’ - GAGCCCGCAGTCGTACGAG - 3’	R: 5’ – CTAGTGGGACGCGGACAT - 3’	1
	CAT-R: 5’ - GGTCTTCGTGGCTGTTTTCT - 3’	2
	Ex2-R: 5’ – CACGCCCTGTTTCTTTGAAT - 3’	3
