# Supplementary material for: TWIST1 Gene: First Insights in Felis catus
Source: Curr Genomics. 2010 May;11(3):212–20. doi: 10.2174/138920210791110933 (PMC2878985; doi:10.2174/138920210791110933)
Supplement: Supplementary file 1 — Supplementary material is available on the publishers Web site along with the published article. [file CG-11-212_SD1.pdf]

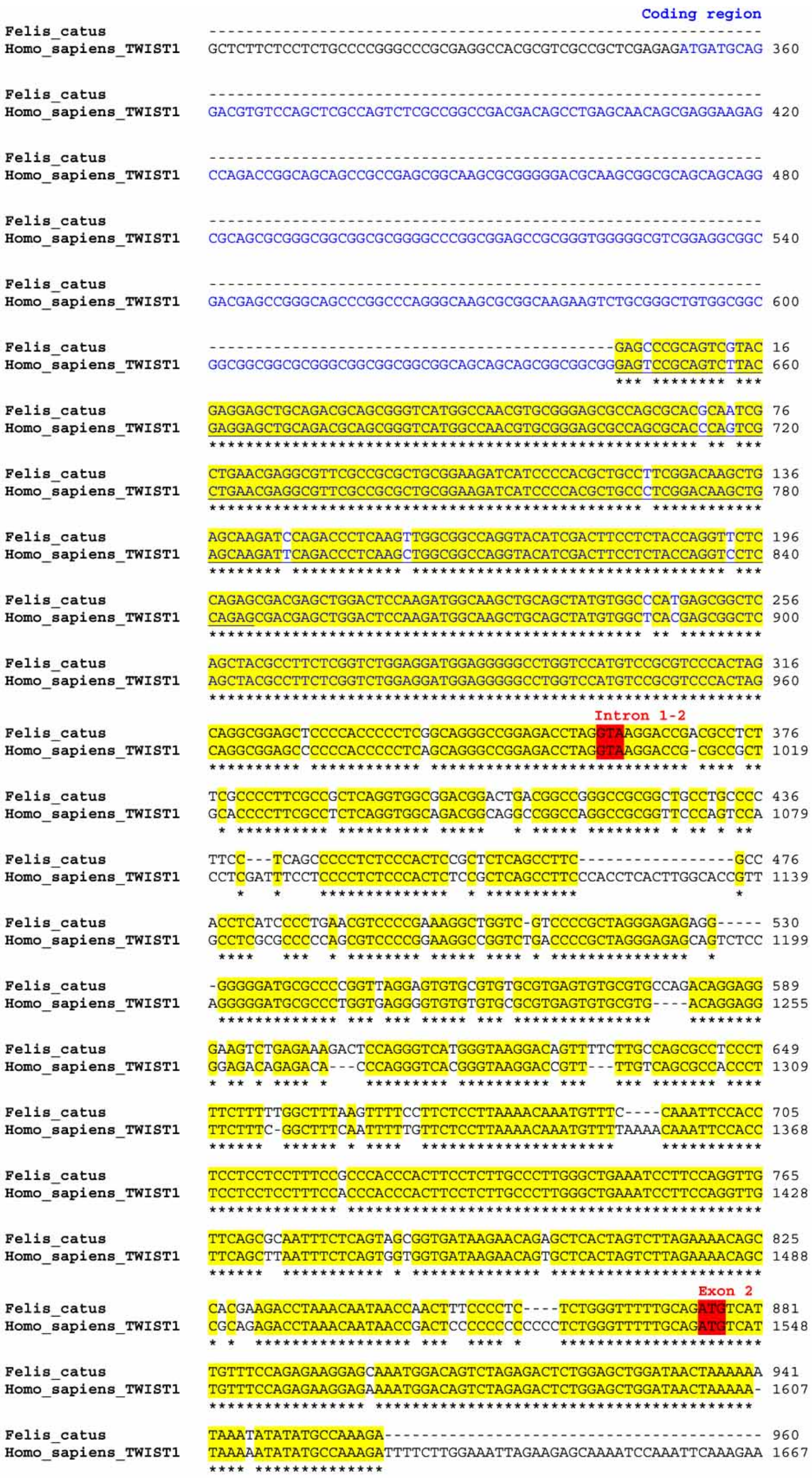

Supplementary Fig. (1). Using the ClustalW program we aligned the 960 bp sequence isolated in *Felis catus* with the similar region of the *TWIST1* gene in *Homo sapiens*. Exon 1, coding region, intron and exon 2 are evidenced. Underline is the sequence amplified by RT-PCR. We can observe major genomic differences in the intronic region. Conversely, there is a high similarity between the sequences of both exons. We can also conclude that the cDNA sequence, obtained by RT-PCR, totally matches the predicted coding region of the *Felis catus TWIST1* gene, suggesting the existence of, at least, one transcript.
